# Characteristics and Mechanisms of Asphalt–Filler Interactions from a Multi-Scale Perspective

**DOI:** 10.3390/ma13122744

**Published:** 2020-06-17

**Authors:** Wenyi Xu, Xin Qiu, Shanglin Xiao, Haojue Hong, Feng Wang, Jie Yuan

**Affiliations:** 1 College of Engineering, Zhejiang Normal University, Jinhua 321004, China; wenyixu@zjnu.edu.cn (W.X.); sl-xiao@zjnu.cn (S.X.); honghaojue@zjnu.edu.cn (H.H.); 2 Key Laboratory of Urban Rail Transit Intelligent Operation and Maintenance Technology & Equipment of Zhejiang Province, Zhejiang Normal University, Jinhua 321004, China; 3 Ingram School of Engineering, Texas State University, San Marcos, TX 78666, USA; f_w34@txstate.edu; 4 Key Laboratory of Road and Traffic Engineering of the Ministry of Education, Tongji University, Shanghai 201804, China; yuanjie@tongji.edu.cn

**Keywords:** asphalt mastic, asphalt–filler interaction, macro-rheological behavior, micromorphology, grey relational analysis

## Abstract

Asphalt mastic plays an important role in asphalt mixtures for pavement engineering. Understanding the asphalt–filler interaction behavior is essential to improve the pavement performance of asphalt mastics. The objective of this paper was to evaluate the asphalt–filler interaction ability based on macro-rheological measurements and to investigate the asphalt–filler interaction mechanism associated with microstructural characteristics of asphalt mastics. First, the asphalt–filler interaction was characterized using macro-rheological features of asphalt mastics based on dynamic shear rheometer (DSR) tests. Second, the physico-chemical interaction between the asphalt and filler was qualitatively evaluated using a Fourier transform infrared (FTIR) spectrometer. Third, the asphalt–filler interaction behavior was investigated in terms of the micro-morphological properties of mineral fillers and asphalt mastics by conducting scanning electron microscopy (SEM) and atomic force microscope (AFM) tests. Finally, the grey relational analysis (GRA) was employed to identify the correlation between the properties of mineral fillers and the macro–micro performances of asphalt mastics. The results show that a higher content of alkaline mineral filler within the critical volume fraction range produced a greater interaction ability between the asphalt and filler. The asphalt–filler interaction is mainly a physical action since no obvious new adsorption peaks appeared in the FTIR spectrum. The micro-morphological characteristics of asphalt mastic mainly depended on the adsorption effect of mineral fillers on polar fractions and the dispersion effect of mineral fillers on wax crystals in the asphalt binder. Based on the results of the GRA, the acidity and content of mineral fillers exhibited a great influence on the micro-morphological and macro-rheological characteristics of asphalt mastics, and the specific surface area of the mineral filler exerted a significant influence on the asphalt–filler interaction ability. Furthermore, the *K-B-*δ index was more appropriate for evaluating the asphalt–filler interaction ability.

## 1. Introduction

During the long-term service period of asphalt pavement, severe distress related to repeated traffic loads, freeze–thaw cycles, and other external factors lead to a significant reduction in the serviceability of asphalt pavement [1]. As stated in asphalt mortar theory, an asphalt mixture is a kind of dispersion system with a multi-level spatial network structure, which may include a coarse dispersion system (asphalt mixture), a subdivided dispersion system (asphalt mortar), and a differential dispersion system (asphalt mastic) [2]. In an asphalt mixture, coarse aggregate as the dispersed phase is distributed in asphalt mortar. As for asphalt mortar, fine aggregate as the dispersed phase is scattered in asphalt mastic, which consists of mineral filler and asphalt binder. Asphalt mastic plays a crucial role in bonding aggregates and filling voids for asphalt mixtures. Many studies have shown that the interaction between asphalt binder and mineral filler (asphalt–filler interaction) would affect the viscoelastic properties and mechanical behaviors of asphalt mastics, which further impose great impacts on the overall pavement performance of asphalt mixtures, such as fatigue damage resistance, low-temperature cracking resistance, high-temperature stability, and moisture stability [3,4]. For example, fatigue damage usually occurs within asphalt mastics due to cohesive failures, from which microcracks initiate and then gradually propagate to form macrocracks. Therefore, evaluating the asphalt–filler interaction ability, exploring related influential factors of asphalt–filler interactions, and revealing the interaction mechanism associated with microstructural characteristics of asphalt mastics are of great significance to better understanding the performance of asphalt mastics and effectively reducing pavement distress.

Various research studies indicated that the performance improvement of an asphalt binder using a mineral filler would mainly depend on mechanical reinforcement and physico-chemical interaction. Volume filling and particle structuralization are two means of mechanical reinforcement [5,6,7]. With a lower mineral filler content, asphalt mastic as a dilute suspension exhibits a moderate improvement of stiffness since filler particles replace parts of the asphalt binder. With the increase in filler content, filler particles begin to aggregate and then constitute an interconnected network at a filler volume fraction of approximately 40%, which leads to a rapid increase in the stiffness of asphalt mastics toward particle structuralization. The physico-chemical interaction is believed to result from the adsorption of polar fractions of asphalt binder onto the surfaces of filler particles [5,8], which leads to the formation of a structural asphalt layer, consequently improving the engineering performance of asphalt mastics.

Numerous studies have shown that the asphalt–filler interaction is susceptible to temperature, loading frequency, filler acidity, filler content, filler particle size, etc. Fan et al. found that temperature had a positive impact on the interfacial adhesive ability of asphalt mastics [9]. Zhang et al. demonstrated the ability of the asphalt–filler interaction decreased with the increase of loading frequencies [10]. Zhang et al. confirmed that the ability of the asphalt–filler interaction is related to the filler acidity, filler particle size, and filler content, in which the parameter of filler particle size would affect the asphalt–filler interaction more than other factors [11]. Antunes et al. concluded that the specific surface area, shape, and texture of mineral filler particles are sensitive factors that influence asphalt–filler interaction behaviors [12]. Some studies indicated that the mechanical performance of asphalt mastics could be improved by increasing the mass ratio of filler to asphalt binder [13] but should be less than 1.5 [14]. Faheem et al. demonstrated that when the volume fraction of a mineral filler is less than 40%, the particle size of fillers plays a significant role in the asphalt–filler interaction ability [15]. Tan et al. concluded that with a finer filler or a higher temperature, the asphalt–filler interaction ability becomes stronger [16]. Romeo et al. demonstrated the interaction between styrene-butadiene-styrene (SBS)-modified asphalt binder and fillers of high fractional voids weaken the fatigue performance and fracture resistance of asphalt mixtures [17]. Moraes et al. indicated that the asphalt–filler interaction could be affected by the filler type and content, which could reduce the effect of aging on the mechanical properties of asphalt mastics [18]. Therefore, these studies confirmed that the basic physical properties of mineral fillers and external environmental factors exert a significant impact on the asphalt–filler interaction.

In addition, rheological-based evaluations are also widely employed to investigate the asphalt–filler interaction ability. Tan et al. utilized the complex modulus coefficient ($\Delta G^{*})$ to evaluate the asphalt–aggregate interaction ability but found that $\Delta G^{*}$ was unable to distinguish between the effect of aggregate powder types on the interaction ability [19]. Guo evaluated the asphalt–filler interaction ability based on the intrinsic viscosity ([η]), and indicated that the intrinsic viscosity was not sensitive enough to distinguish between the interaction abilities of different filler volume fractions [20]. Tan et al. demonstrated that the complex viscosity coefficient ($\Delta \eta^{*}$) could be employed to evaluate the asphalt–filler interfacial interaction ability but was not susceptive to filler types [21]. Nelson et al. applied the Einstein coefficient $K_{E}$ to analyze the enhancement effect of glass spheres on the modulus of particulate-filled composites [22], whereas Zhang et al. found that $K_{E}$ was inapplicable toward analyzing the effect of filler concentrations [23]. Ziegel et al. established two indexes, namely, *K-B-*$G^{*}$ and *K-B-δ*, to explore the interaction behaviors between polymer and filler by considering the particle size, density, structure, and voids of inorganic filler [24,25]. Kubat et al. utilized the *L-A-δ* index to evaluate the interaction between the matrix phase and the reinforcement phase based on the change of the mechanical loss factor [26]. Zhang et al. investigated the validity of using $\Delta G^{*}$, $K_{E}$, and *K-B-*$G^{*}$ for evaluating the asphalt–filler interaction ability, where the results indicated that *K-B-*$G^{*}$ could better distinguish the effect of filler content on the change in asphalt–filler interaction behavior [27]. Tan et al. analyzed the sensitivity of *K-B-δ*, *L-A-δ*, $\Delta G^{*}$, and $\Delta \eta^{*}$, and concluded that *K-B-δ* exhibited a higher applicability than the other indexes [28]. Liu et al. utilized four indexes, namely, $\Delta G^{*}$, *K-B-*$G^{*}$, *L-A-δ*, and *K-B-δ*, to assess the effects of temperature and loading frequency on the asphalt–filler interaction and found that the sensitivity of the *K-B-δ* index was the highest of the indexes under the condition of less than 40% volume fraction [29]. Liu et al. compared the effectiveness of several indexes, including $\Delta G^{*}$, *L-A-δ*, *K-B-δ*, and *K-B-*$G^{*}$, for evaluating the asphalt–filler interaction ability and demonstrated that *K-B-δ* was the most appropriate index among them, displaying the highest sensitivity within the critical volume fraction range [30]. As aforementioned, these studies greatly contributed to the research on asphalt–filler interaction evaluation and gave insights into interpreting the characteristics and behaviors of the asphalt–filler interaction.

Recent efforts focused on the application of microscopic techniques have yielded significant outcomes in understanding the microstructural characteristics of asphalt binders. Loeber et al. investigated the interaction between polymer and asphalt binders by observing microstructural changes of asphalt binders and found that the aggregation of asphaltene could be clearly observed using a fluorescence microscope (FM), scanning electron microscope (SEM), or atomic force microscope (AFM) [31]. Tan et al. utilized Fourier transform infrared (FTIR) spectroscopy to describe the physico-chemical interaction between an asphalt binder and filler, and indicated that the asphalt–filler interaction was mainly a physical process [32]. By using light microscopy, FM, and SEM, López et al. proposed that the reason for the increase of viscosity of a rubber-modified asphalt binder was the adsorption of rubber particles for bitumen components [33]. Fischer et al. employed AFM to determine the contact angles between mineral filler particles and asphalt binders to account for the asphalt–filler interaction ability and indicated that the adsorption effect of mineral particles on polar fractions in asphalt binder leads to the aggregation of asphaltene onto the surface of filler particles [34]. Nazzel et al. demonstrated that the interaction between nanoclay and asphalt binder fractions results in the improvement of material stiffness by analyzing the microstructural characteristics and mechanical behaviors of asphalt mastic [35]. Davis et al. demonstrated that the adsorption of mineral filler for polar fractions in an asphalt binder would change the micro-morphologies of asphalt binder and further affect the macro-rheological properties of asphalt mastics [36]. Using AFM, Li et al. discovered that the more polar filler particles would strengthen the asphalt–filler interaction and lead to a larger adhesion capacity between them [37]. Guo et al., with the help of AFM, indicated that the adsorbed asphalt binder exhibits a higher modulus due to the asphalt–filler interaction [38]. Saha et al. indicated that the addition of fly ash could change the morphology of micelle structures, which would improve the stiffness of an asphalt binder [39]. Based on AFM, Lv et al. indicated that the surface micro-morphological features of mineral particles affects the adhesion effect between asphalt binders and mineral particles [40]. The microscopic techniques, such as SEM, AFM, and FTIR, are certainly helpful for investigating the effect of mineral fillers on the microstructural characteristics of asphalt mastics.

In summary, previous studies have enhanced our understanding of the viscoelastic properties and mechanical behaviors of asphalt mastics. However, to date, little attention has been given to what extent and how the microstructural characteristics would affect the behaviors of the asphalt–filler interaction. A further understanding of the asphalt–filler interaction through a rheological-morphological coupling method from a multi-scale perspective is important to promote the engineering performance of asphalt mastics in the design process of asphalt mixtures. Therefore, the objectives of this study were to evaluate the asphalt–filler interaction ability based on macro-rheological measurements, to investigate asphalt–filler interaction mechanisms associated with microstructural characteristics of asphalt mastics, and then to explore the relationship between the properties of mineral fillers and the macro–micro performances of asphalt mastics. Specifically, this study aimed to:

1. Investigate the macro-rheological behaviors of asphalt mastic by considering different types and contents of mineral fillers, and to evaluate the asphalt–filler interaction ability by selecting appropriate macro-rheological indexes.
2. Develop the characteristics and mechanisms of the asphalt–filler interaction through physico-chemical interaction analysis based on FTIR and micro-morphological characteristics observation based on SEM and AFM tests.
3. Establish a grey relational analysis model to quantitatively evaluate the influence of mineral fillers on macro–micro properties of asphalt mastics and the asphalt–filler interaction ability.

## 2. Materials and Test Methods

### 2.1. Materials

#### 2.1.1. Asphalt Binder and Mineral Filler

A 70 penetration grade base asphalt binder (SINOPEC Zhenhai Refining & Chemical Company, Ningbo, China) was selected in this study, where its basic properties are shown in Table 1.

Three kinds of mineral fillers (passing the 0.075 mm sieve), namely, limestone, diabase, and granite, were selected for the asphalt mastic preparation. To investigate the effect of filler acidity on the asphalt–filler interaction, the SiO_2_ contents of the fillers were determined using an X-ray spectrometer (D8 Advance, Bruker AXS, Karlsruhe, Germany). The specific surface area (SSA) of each filler was obtained by applying the Brunauer–Emmett–Teller (BET, Nova4000e, Quantachrome, Boynton Beach, FL, USA) method [41]. The properties of the mineral fillers are shown in Table 2. It is believed that aggregates with more than 65% SiO_2_ are acidic materials, aggregates with 52–65% SiO_2_ are intermediate materials, and those with less than 52% are alkaline materials [42]. Therefore, the ranking of filler acidity based on the X-ray result was granite > diabase > limestone.

#### 2.1.2. Asphalt Mastic

For the asphalt mastic preparation, the mineral filler and asphalt binder were preheated separately at 150 °C for 2 h. Then, a specific amount of filler was slowly added into the base asphalt binder at mass ratios (filler to asphalt, F/A) of 0.8, 1.0, and 1.2, which are less than the critical volume fraction of 40% to avoid particle structuralization [15,23]. The blending process was conducted using a high-shear mixer (EA300-H, Shanghai, China) at 150 °C at 1000 rpm for 30 min to obtain a homogeneous asphalt mastic. After mixing, the blends were divided into small aluminum cans and stored in a sealed container for subsequent tests. The basic properties of the three asphalt mastics are shown in Table 3, in which LAM, DAM, and GAM denote to limestone asphalt mastics, diabase asphalt mastics, and granite asphalt mastics, respectively.

### 2.2. Test Methods

#### 2.2.1. Dynamic Shear Rheometer

The asphalt–filler interaction was determined using the rheological characteristics of asphalt mastics. The frequency sweep tests were used to find the characteristics of the complex shear modulus ($\left|G^{*}\right|$) and phase angle (δ) of asphalt mastics with a dynamic shear rheometer (DSR) apparatus (Anton Paar MCR 302, Graz, Austria). In the DSR tests, a parallel plate with an 8 mm diameter and 2 mm gap was chosen. The testing temperature was 25 °C and the frequency of loading was from 10 Hz to 0.01 Hz with a control strain of 5%. Two replicates were performed in each case to ensure that the results had an acceptable repeatability.

#### 2.2.2. Fourier Transform Infrared Spectroscopy

The microscopic Fourier transform infrared (FTIR) spectroscopy (Nicolet iS5, Thermo Fisher Scientific, Waltham, MA, USA) was used to analyze the compositions of the asphalt materials. During the tests, the attenuated total reflectance (ATR) approach was utilized for the base asphalt binder and asphalt mastics, and the thin potassium bromide (KBr) disk method was employed for the mineral fillers. The spectra were collected over the wavenumber range of 4000 to 600 cm^−1^ with a resolution of 4 cm^−1^. The spectrum of each sample was an average of 16 spectra. The types of functional groups were identified by observing the position, shape, and width of the FTIR spectrum peak, and the content of each group was evaluated according to the intensity of the spectral peak to evaluate the physico-chemical interaction process between the asphalt binder and mineral filler.

#### 2.2.3. Scanning Electron Microscopy

The scanning electron microscopy (SEM) apparatus (COXEM EM30-AX Plus, Daejeon, Korea) was employed to observe the micromorphology of the mineral fillers and the interfacial morphology of the asphalt filler. SEM samples with a dimension of 25 mm × 35 mm were coated with a thin layer of palladium using low vacuum sputtering. Three surface locations of each sample were scanned at 20 kV with various magnifications of 2000×, 2500×, 3000×, and 5000×. Three replicates were scanned in each test and a representative sample was selected for the analysis.

#### 2.2.4. Atomic Force Microscopy

An atomic force microscopy (AFM) apparatus (Bruker Dimension ICON, Germany) was used to observe the phase structure morphology of the asphalt mastic. All images were obtained using the peak force tapping mode. The type of probe was a SCANASYST-AIR with a characteristic resonance frequency of 80 kHz. The micro-cantilever with an elastic constant of 0.4 N/m and length of 600 nm was used. The testing scanning frequency was 1 Hz and the image scanning area was 20 μm × 20 μm. The samples of asphalt mastic for the measurement were prepared with a heat-cast method [43]. First, a bead of asphalt mastic was dropped onto the glass slide, which was heated for 10 min in the oven at 120 °C. Second, the thin film was placed on the homogenizer and rotated at a speed of 5000 rad/min for 1 min to ensure that the testing mastic was evenly distributed on the surface of the glass slide. Then, the asphalt-binder-coated glass slide was put back in the oven for 5 min to obtain a smooth surface. Finally, the sample was placed in room temperature to cool for 24 h. All samples were observed at a room temperature of 25 °C. Two different surface locations of each sample were scanned, three replicates were conducted to ensure the consistency and reliability of testing results, and a representative result was used for the analysis. The images were analyzed using the NanoScope Analysis software 1.40 (Bruker AXS, Karlsruhe, Germany) that match the AFM apparatus to obtain the related morphological parameters of the asphalt mastics.

#### 2.2.5. Grey Relation Analysis

The grey relation analysis (GRA) was utilized to investigate the correlation between the properties of the mineral fillers and the macro–micro performances of the asphalt mastics. The GRA is a factor analysis method used to solve problems of complicated interrelationships between multiple factors and variables by defining the reference sequences and comparative sequences [44,45,46]. To perform the GRA, three steps are performed, namely, data pre-processing, grey relational coefficient determination, and grey relational grade calculation.

Assuming that the reference sequences and comparative sequences are represented as $x_{0}^{\left(0\right)}\left(k\right)$ and $x_{i}^{\left(0\right)}\left(k\right)$, respectively. The original data series $X^{\left(0\right)}\left(k\right)$ can be expressed in the form of Equation (1):

(1) $$X^{\left(0\right)}(k)=\left[\begin{array}{c} x_{0}^{\left(0\right)}\left(k\right)\\ x_{i}^{\left(0\right)}\left(k\right) \end{array}\right]=\left[\begin{array}{cccc} x_{0}^{\left(0\right)}\left(1\right) & x_{0}^{\left(0\right)}\left(2\right) & \ldots & x_{0}^{\left(0\right)}\left(n\right)\\ x_{1}^{\left(0\right)}\left(1\right) & x_{1}^{\left(0\right)}\left(2\right) & \ldots & x_{1}^{\left(0\right)}\left(n\right)\\ x_{2}^{\left(0\right)}\left(1\right) & x_{2}^{\left(0\right)}\left(2\right) & \ldots & x_{2}^{\left(0\right)}\left(n\right)\\ \vdots & \vdots & \vdots & \vdots \\ x_{m}^{\left(0\right)}\left(1\right) & x_{m}^{\left(0\right)}\left(2\right) & \ldots & x_{m}^{\left(0\right)}\left(n\right) \end{array}\right],$$

where *i* = 1, 2, …, m; k = 1, 2, …, n; m represents the number of factors; and n denotes the number of data items.

(1) Data pre-processing

Data pre-processing is used to transfer the original sequences into comparable sequences. In this study, the commonly used normalization method was adopted to divide the original sequence by the first value of the sequence, as shown in Equations (2) and (3):

(2) $$x_{0}^{*}=\frac{x_{0}^{\left(0\right)}\left(k\right)}{x_{0}^{\left(0\right)}\left(1\right)},$$

(3) $$x_{i}^{*}=\frac{x_{i}^{\left(0\right)}\left(k\right)}{x_{i}^{\left(0\right)}\left(1\right)},$$

where $x_{0}^{*}$ and $x_{i}^{*}$ are the reference sequences and the comparative sequences after data pre-processing, respectively.

(2) Grey relational coefficient

Following the data pre-processing, the grey relational coefficient is determined from the normalized sequences to estimate the relationship between the reference sequences and the comparative sequences. The grey relational coefficient is calculated using Equation (4):

(4) $$\gamma_{0i}(k)=\frac{\Delta_{\min}{+\xi \Delta }_{\max}}{\Delta_{0i}{(k)+\xi \Delta }_{\max}},$$

where $\gamma_{0i}\left(k\right)$ denotes the grey relational coefficient between the $x_{0}^{*}\left(k\right)$ and $x_{i}^{*}\left(k\right)$, $\Delta_{0i}\left(k\right)$ is the deviation sequence of $x_{0}^{*}\left(k\right)$ and $x_{i}^{*}\left(k\right)$, $\Delta_{0i}(k)=\left|x_{0}^{*}\left(k\right)-x_{i}^{*}\left(k\right)\right|$, $\Delta_{\min}{=\min}_{i}\min_{k}\Delta_{0i}\left(k\right)$, $\Delta_{\max}{=\max}_{i}\max_{k}\Delta_{0i}\left(k\right)$, and ξ∈[0,1] is the distinguishing coefficient (0.5 is generally used).

(3) Grey relational grade

After the grey relational coefficient is derived, the grey relational grade $\Gamma_{0i}$ can be calculated using Equation (5); the higher value of the grey relational grade, the stronger the relational degree between the reference sequence and the given comparative sequence:

(5) $$\Gamma_{0i}=\frac{1}{n}\sum_{k=1}^{n}\gamma_{0i}\left(k\right).$$

## 3. Results and Discussion

### 3.1. Asphalt–Filler Interaction Ability Evaluation

#### 3.1.1. Analysis Index

An asphalt mastic can be regarded as a typical multi-phase blend. The viscoelastic properties and mechanical behaviors of an asphalt mastic are affected by the asphalt–filler interaction, which is sensitive to the particle size, distribution, content and the acidity of mineral fillers, etc. Therefore, an accurate understanding of the asphalt–filler interaction behavior would provide support for controlling mineral filler addition to ensure good performance of asphalt mastics.

Based on previous studies, the *K-B-*δ index and *K-B-*$G^{*}$ index were shown to be more appropriate for evaluating the asphalt–filler interaction ability [10,19,27,30]. The *K-B-*δ index and *K-B-*$G^{*}$ index are shown in Equations (6) and (7) [24,25], respectively:

(6) $$K\text{-}B\text{-}\delta =\frac{\left({\tan\delta }_{m}/{\tan\delta }_{c}\right)\;-\;1}{{1.5\;\varphi }_{f}},$$

where $\delta_{c}$ is the phase angle of asphalt mastics (°), $\delta_{m}$ is the phase angle of base asphalt binder (°), and $\varphi_{f}$ is the volume fraction of mineral fillers (%). The greater the value of *K-B-*δ, the stronger asphalt–filler interaction ability.

(7) $$K{\text{-}B\text{-}G}^{*}=\frac{\left(G_{c}^{*}/G_{m}^{*}\right)\;-\;1}{\left({1.5+G}_{c}^{*}/G_{m}^{*}\right){.\varphi }_{f}},$$

where $G_{c}^{*}$ is the complex modulus of asphalt mastics and $G_{m}^{*}$ is the complex modulus of the base asphalt binder. The greater the value of *K-B-*$G^{*}$, the stronger asphalt–filler interaction ability.

The volume fraction ($\varphi_{f}$) is an important parameter for calculating *K-B-*δ and *K-B-*$G^{*}$. It can be obtained from Equation (8) [47]:

(8) $$\varphi_{f}=\frac{V_{f}}{V_{a}{+V}_{f}}=\frac{m_{f}/\rho_{f}}{m_{a}/\rho_{a}{+m}_{f}/\rho_{f}}=\frac{\left(m_{f}/m_{a}\right)/\rho_{f}}{1/\rho_{a}+\left(m_{f}/m_{a}\right)/\rho_{f}},$$

where $V_{f}$ is the filler volume; $V_{a}$ is the asphalt binder volume; $m_{f}$ is the filler mass; $m_{a}$ is the asphalt binder mass; $\rho_{f}$ is the filler density, as given in the Table 2; and $\rho_{a}$ is the asphalt binder density, which is approximately equal to 1.030 g/cm^3^.

#### 3.1.2. Evaluation Results

**Figure 1:**
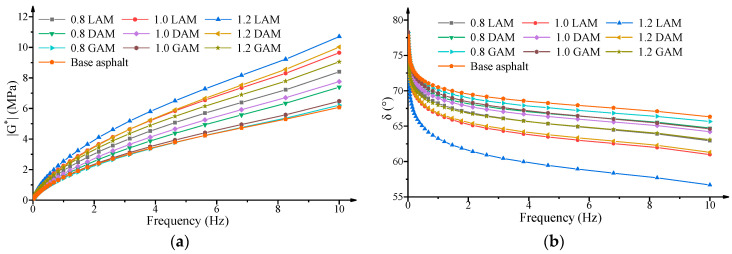
Frequency sweep curves for the base asphalt binder and asphalt mastics. LAM: limestone asphalt mastic, DAM: diabase asphalt mastic, GAM: granite asphalt mastic. (**a**) $\left|G^{*}\right|$ versus frequency; (**b**) δ versus frequency.

Figure 1 shows the curves of the complex shear modulus ($\left|G^{*}\right|$) and phase angle (δ) of the base asphalt binder and asphalt mastics. As shown in Figure 1a, the asphalt mastics LAM, DAM, and GAM presented larger values for $\left|G^{*}\right|$ than the base asphalt binder over the full frequency range. For any of three asphalt mastics, the value of $\left|G^{*}\right|$ rapidly improved with the increase of the F/A ratio. Under the condition of the same F/A ratio, the $\left|G^{*}\right|$ of LAM presented the highest value, followed by DAM, GAM, and the base asphalt binder. In contrast, Figure 1b presents an opposite trend showing that all asphalt mastics exhibited lower values of δ than that of base asphalt binder at the same F/A ratio and frequency. The ranking of δ is listed as base asphalt binder > GAM > DAM > LAM. Furthermore, the value of δ decreased with an increase of filler content. Furthermore, $\left|G^{*}\right|$ increased and δ decreased with a loading frequency increase for all asphalt mastics. Based on the results of $\left|G^{*}\right|$ and δ, it is believed that the filler played a significant role in changing the viscoelastic properties and improving the mechanical performance of the asphalt binders [48,49].

**Figure 2:**
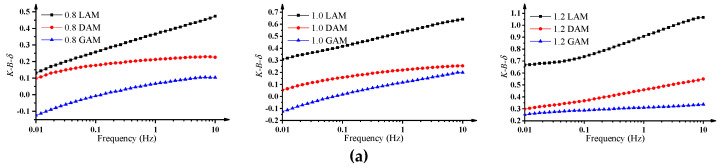

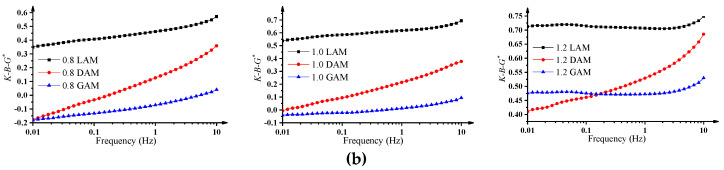
Change trends of *K-B-*δ and *K-B-*$G^{*}$ of three asphalt mastics with different F/A ratios and loading frequencies. (**a**) *K-B-*δ curves frequency; (**b**) *K-B-*$G^{*}$ curves frequency.

**Figure 3:**
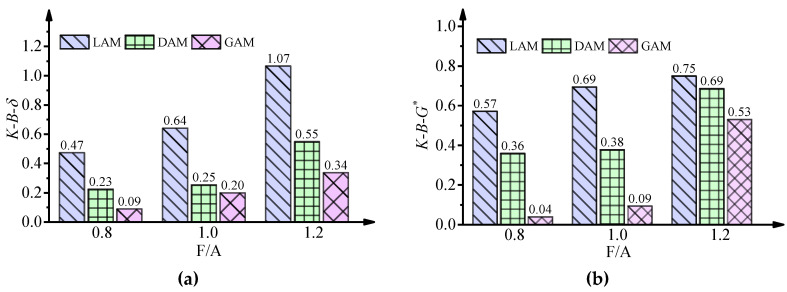
Maximum values of *K-B-*δ and *K-B-*$G^{*}$ of three asphalt mastics with different F/A ratios. (**a**) *K-B-*δ curves F/A; (**b**) *K-B-*$G^{*}$ curves F/A.

Based on the results of $\left|G^{*}\right|$ and δ, the change curves of *K-B-*δ and *K-B-*$G^{*}$ for the three asphalt mastics over the full frequency range are shown in Figure 2. The results indicate that the values of *K-B-*δ and *K-B-*$G^{*}$ presented an increasing trend with the increase of loading frequency, demonstrating that the asphalt–filler interaction ability is susceptible to loading frequencies. The maximum values of *K-B-*δ and *K-B-*$G^{*}$ occurred at the frequency of 10 Hz; the maximum values of *K-B-*δ and *K-B-*$G^{*}$ of three asphalt mastics with different F/A ratios are presented in Figure 3. The results demonstrate that the *K-B*-δ and *K-B-*$G^{*}$ of LAM presented the highest value, followed by DAM and GAM, under the condition of the same F/A ratio, which could also be inferred as showing that the interaction ability between the alkaline filler and asphalt binder was stronger. Furthermore, the values of *K-B-*δ and *K-B-*$G^{*}$ rapidly improved with the increase of the F/A ratio for all three asphalt mastics and achieved the maximum value when F/A was equal to 1.2, which indicates that a higher F/A ratio promoted the asphalt–filler interaction ability. Based on the above results, it is believed that the asphalt–filler interaction ability is closely related to the macro-rheological properties of asphalt mastics. The indexes *K-B-*δ and *K-B-*$G^{*}$ were appropriate for evaluating the asphalt–filler interaction ability and could effectively distinguish the effect of the F/A ratio and filler type on the asphalt–filler interaction ability. Furthermore, it is worth mentioning that deeply understanding the asphalt–filler interaction mechanism associated with microstructural characteristics of asphalt mastics would make a great contribution to improving the engineering performance of asphalt mastics.

### 3.2. Micro-Structural Characteristics of Asphalt Mastics

#### 3.2.1. FTIR

**Figure 4:**
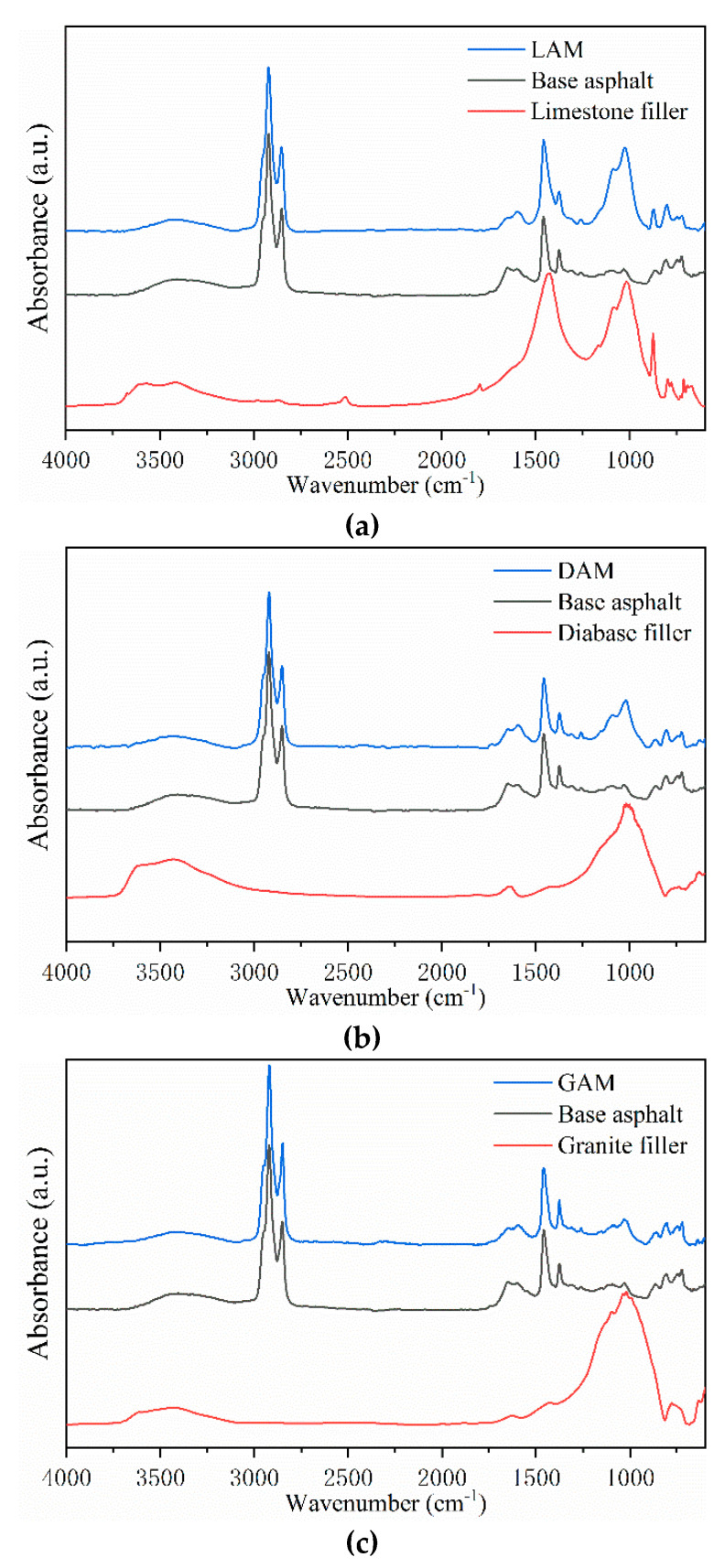
Infrared spectrum of the base asphalt binder, fillers, and asphalt mastics. (**a**) Limestone; (**b**) Diabase; (**c**) Granite.

The spectra of the base asphalt binder, mineral fillers, and asphalt mastics with an F/A ratio of 1.0 are shown in Figure 4. For all spectra, there were large and broad bands found near 3615 cm^−1^ and 3419 cm^−1^, which was ascribed to the stretching vibration of hydroxyl (O–H). In terms of the base asphalt binder, the peak from 3100 to 2754 cm^−1^ was the stretching vibration of C–H. The small peak at 1600 cm^−1^ was caused by the stretching vibration of C=C. The C–H bonds observed within the 1462–1376 cm^−1^ region were classified as the in-plane bending vibrations of CH_2_ and CH_3_. The peak near 723 cm^−1^ corresponded to the rocking vibration of (–CH_2_–)_n_, (n > 4). As for the filler, the peaks near 1019 cm^−1^, 1160 cm^−1^, and 770 cm^−1^ for different fillers indicated the existence of SiO_2_. Limestone filler not only displayed most of the absorption peaks of the diabase filler and granite filler but also had its own absorption peaks. For example, the strong absorption peak at 1440 cm^−1^, and the medium peak at 875 cm^−1^, the small peaks at 1080 and 713 cm^−1^ indicated that there was an obvious CaCO_3_ structure in the limestone filler. As for the asphalt mastics, most of the absorption peaks were the superposition of the base asphalt binder and filler, especially for the change of the absorption peak of 1019 cm^−1^. No obvious new peak can be observed in Figure 4, which indicates that the asphalt–filler interaction was mainly caused by physical action. Our findings share some similarities with previous results reported in the literature [32,50].

#### 3.2.2. SEM

**Figure 5:**
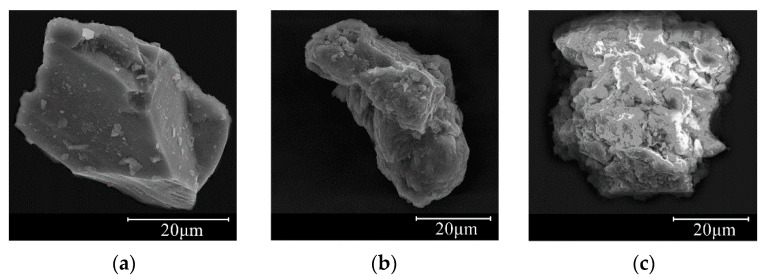
Micro surface texture morphologies of the mineral filler particles. (**a**) Granite filler (5000×); (**b**) Diabase filler (3000×); (**c**) Limestone filler (2500×).

The micro surface texture properties of mineral filler particles are the important factors that affect the interfacial interaction between the filler and asphalt binder [51]. In this study, the surface micro-morphological characteristics of the three studied mineral filler particles were observed using an SEM, as presented in Figure 5. The results indicate that the micro surface texture of the limestone filler particle was more rough and uneven due to the existence of rich folds and protrusions compared with the granite filler and diabase filler, which could increase the asphalt–filler interfacial adhesive force. It is worth mentioning that a similar phenomenon was also discovered by Fischer et al. [34]. Meanwhile, a large number of micropores were also observed on the surface of limestone filler particles, which means that the filler particles could be easily infiltrated through the adsorption and capillarity of the asphalt binder, contributing to the improvement of the adhesion ability between the asphalt binder and limestone filler, as mentioned in Aburkaba and Muniandy [52]. In contrast, granite filler particles with the smoothest surface texture produced the weakest interfacial adhesion among the three studied mineral fillers.

**Figure 6:**
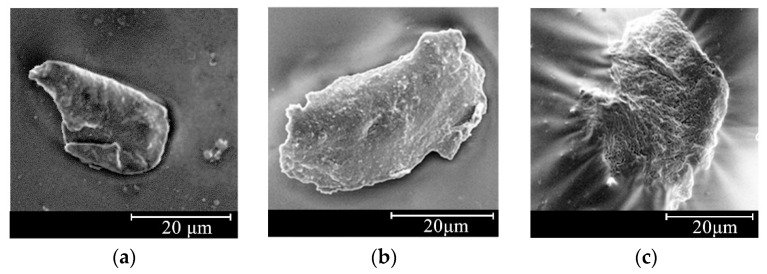
Interfacial morphology of the asphalt mastics. (**a**) GAM (5000×); (**b**) DAM (2000×); (**c**) LAM (2000×).

The adhesion ability between the asphalt binder and filler was also reflected by the interfacial tension state to some extent. The interfacial micrographs obtained using an SEM for LAM, DAM, and GAM in the case of an F/A ratio of 1.0 are shown in Figure 6. As illustrated in Figure 6c, the limestone filler particles were completely immersed in the asphalt binder with an indistinct boundary between the asphalt binder and the limestone filler particles exhibited a wrinkled condition, which indicates that the asphalt binder could provide a better adhesion effect on the limestone filler particles due to the powerful polar absorption ability. Regarding DAM and GAM, a distinct boundary between the asphalt binder and filler particles was observed and there existed an isolated envelope line between them, which indicated that although the asphalt binder provided the wrapping effect on the filler particles, the interfacial bonding interaction between them was weaker than that of LAM. Furthermore, the SSA of the mineral filler is an important parameter influencing the asphalt–filler interaction, as shown by Tan et al. [53]; the SSA value of the limestone filler was the largest among the three mineral fillers, as shown in Table 2, which provided the greatest contact area for bonding with the asphalt binder. Therefore, it was concluded that the adhesive ability between the asphalt binder and limestone filler was the strongest among the three studied asphalt mastics. The following ranking was DAM and GAM.

#### 3.2.3. AFM

**Figure 7:**
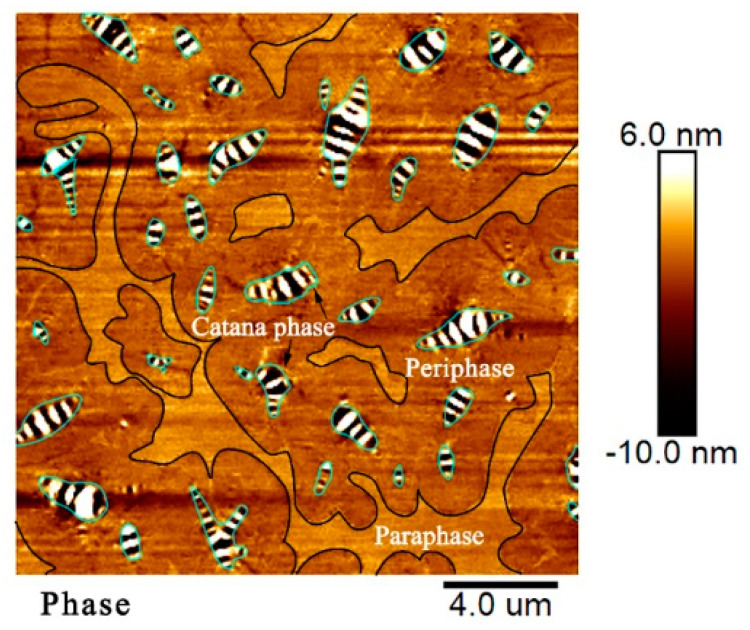
Phase structure of the base asphalt binder.

The micro-morphological characteristic referred to as the phase structure of the base asphalt binder was observed using an AFM, as illustrated in Figure 7, which presents the typical three-phase structure of an asphalt binder. The alternating black and white areas were the catana phases or called “bee structures.” The dark brown area enclosing the bee phase was the periphase. The light brown area neighboring each periphase was the paraphase. The observation presents similar results to many traditional asphalt binders in past investigations [54,55,56]. It is worth mentioning that the formation mechanism of the “bee structure” has received extensive attention in previous studies, which revealed that the existence of the bee phase in an asphalt binder was attributed to the interaction between wax crystals and polar fractions (asphaltene and resin) in the asphalt binder, and the wax crystallization around the polar fractions [57,58,59,60].

**Figure 8:**
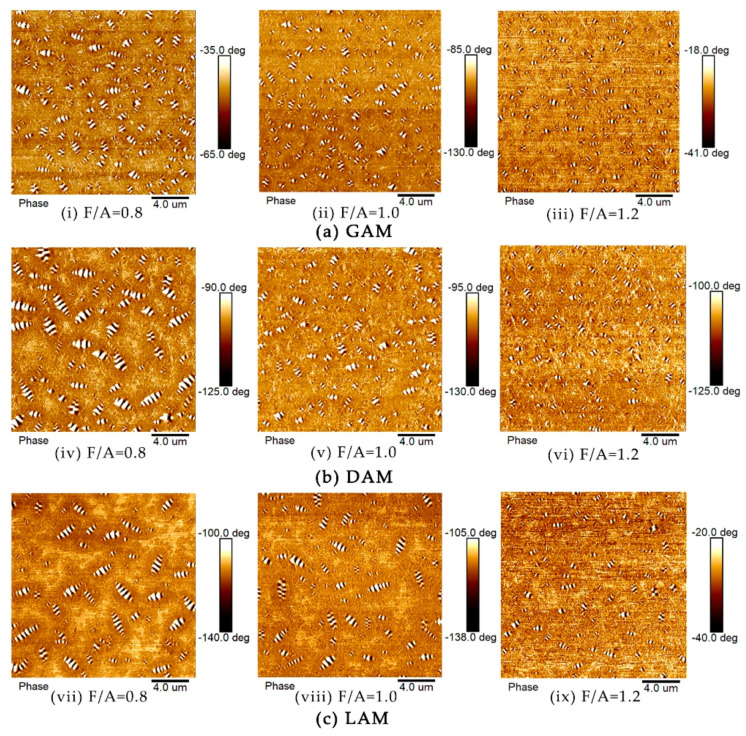
AFM phase morphology images of asphalt mastics. (**a**) GAM; (**b**) DAM; (**c**) LAM.

The mineral filler is a highly polar material with a strong adsorption capacity for polar fractions in an asphalt binder, especially for alkaline limestone fillers, which could strengthen the asphalt–filler interfacial adhesion ability by changing the micro-morphological characteristics of the asphalt binders [61,62]. The AFM phase morphology images of the three asphalt mastics with different F/A ratios are presented in Figure 8. The results show that the size of the “bee structure” is sorted from large to small and by LAM, DAM, and GAM at relatively low F/A ratios. The main reason for this was attributed to the improvement of the adsorption capacity of the filler particles for polar fractions in the asphalt binder with the increase of the alkalinity of mineral fillers, which expedited the accumulation and growth of wax crystals around the polar fractions. Therefore, similar to the “bee structure” size of base asphalt binder in terms of the morphology, the “bee structure” size of the LAM was larger than those of DAM and GAM at with an F/A ratio of 0.8.

Furthermore, with the increase of the F/A ratio, the size of the “bee structure” decreased but the quantity increased for all three asphalt mastics. The “bee structure” in the micromorphology images present a small and dense state. The main reason for this fact is that the dispersive filler particles with the ability to adsorb polar fractions caused the dispersion of wax crystals along with the dispersed polar fractions. In other words, it is believed that mineral fillers could disperse wax crystals and prevented wax crystals from forming large crystal clusters with the increase of the F/A ratio and further resulted in the dispersion of the “bee structure,” which is consistent with the view of Gong et al. [63]. Moreover, as the filler content increased, the dispersion effect of the fillers on wax crystals was gradually enhanced; therefore, the small and dense state of the “bee structure” in the asphalt mastic was clearly observed.

Therefore, it can be concluded that the micro-morphological characteristics of the asphalt mastics mainly depended on the adsorption effect of the mineral fillers on polar fractions and the dispersion effect of the mineral fillers on the wax crystals. Meanwhile, the micro-morphological characteristics of the asphalt mastics would be susceptible to the acidity and content of the mineral fillers. For example, with the increase of alkalinity, the filler particles could make a greater contribution to the adsorption of polar fractions in the asphalt binder, especially with a lower F/A ratio. With the increase of the F/A ratio, the filler particles would have a more significant influence on the dispersion effect on wax crystals in the asphalt binder, regardless of the filler acidity.

Lastly, the phenomenon of the colloidal structure transition was clearly observed, especially for LAM, as presented in Figure 8c. Under the condition of F/A ratios of 0.8 and 1.0, a small number of polar fractions aggregated together to form micelles that dispersed in light fractions of the asphalt binder, which presented a chain-like “sol-gel structure” with a certain strength and good fluidity. When the F/A ratio was 1.2, the polar fractions were more evenly distributed and were better diffused with light fractions due to the adsorption effect of the mineral fillers, which exhibited a network-like “sol-gel structure” with high viscoelasticity, as well as a sufficient fluidity. As mentioned in Li et al. [64], the better the distribution state of the polar fractions, the better the engineering characteristics of the asphalt materials. Therefore, the asphalt mastics with a higher filler content that is less than the critical volume fraction would exhibit better pavement performance when in service.

#### 3.2.4. Quantitative Analysis of the AFM Images

**Figure 9:**
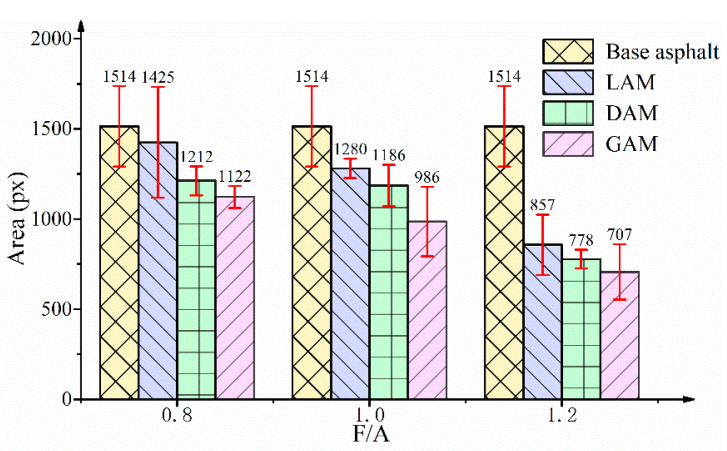
Bee phase area of asphalt mastics.

As mentioned above, the micro-morphological differences of the asphalt mastic are attributable to the asphalt–filler interaction, which is thought to result from the adsorption of filler particles for polar fractions in the asphalt binder [36]. The micro-morphological surface indexes, namely, the bee phase area, surface height, and surface roughness, were extracted from the phase morphology AFM images to quantitatively analyze the micro-morphological differences of different asphalt mastics. Figure 9 presents the results of the bee phase area measured using Image-Pro Plus software 6.0 (Media Cybernetics Corporation, Rockville, MD, USA), in which the bee phase areas of the three asphalt mastics with different F/A ratios were less than that of the base asphalt binder and exhibited a decreasing trend with an increase of the F/A ratio. Furthermore, the bee phase area value of LAM was larger than those of DAM and GAM for all F/A ratio conditions. The results indicated that the bee phase area decreased with the increase of the filler acidity and filler content, which is consistent with the observation results from the AFM images.

**Figure 10:**
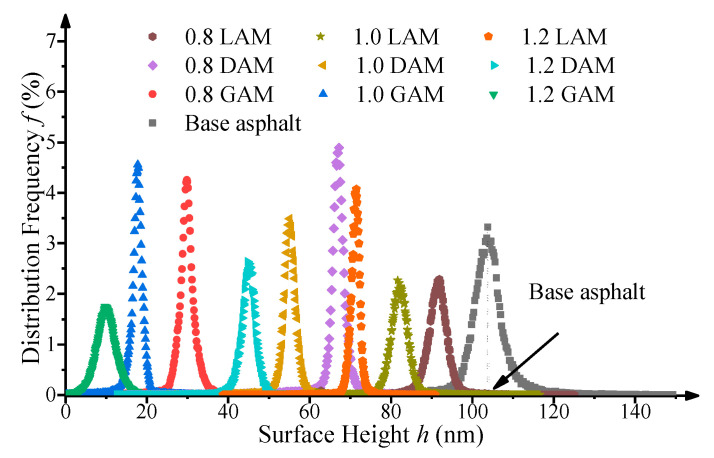
Distribution frequency of the surface height.

Figure 10 shows the distribution frequencies of the phase morphology surface height of the asphalt mastics with different F/A ratios calculated using the NanoScope Analysis software 1.40 (Bruker AXS, Karlsruhe, Germany). As can be seen in Figure 10, the height value presented an approximately normal distribution. In this study, the surface height corresponding to the largest frequency was selected as the representative height of each sample. The surface height of the base asphalt binder was 103 nm, which was larger than those of the three asphalt mastics with different F/A ratios. Under the condition of an F/A ratio of 0.8, the surface heights of LAM, DAM, and GAM were 92 nm, 68 nm, and 30 nm, respectively, which indicated the surface height gradually decreased with the increase of filler acidity. Similar trends were also found with F/A ratios of 1.0 and 1.2. At the same F/A ratio, the height of LAM was 92 nm at F/A = 0.8, 83 nm at F/A = 1.0, and only 72 nm at F/A = 1.2, which demonstrated that the surface height of the asphalt mastic had a negative correlation with the F/A ratio. The variation characteristic of the surface height of DAM and GAM with different F/A ratios was similar to that of LAM.

Furthermore, it has been reported that the level of phase separation could be characterized by the surface roughness through AFM measurements [65]. In this study, the root-mean-square roughness (*Rq*) according to Equation (9) was employed to quantitatively evaluate the surface texture of each sample:

(9) $$Rq=\frac{\oint \oint {\left[h\left(x,y\right)-h_{0}\right]}^{2}dA}{\oint \oint dA},$$

where A is the scanning area, ∮∮dA is the integral of A, h(x,y) is the surface height function, and $h_{0}$ is the reference height.

**Figure 11:**
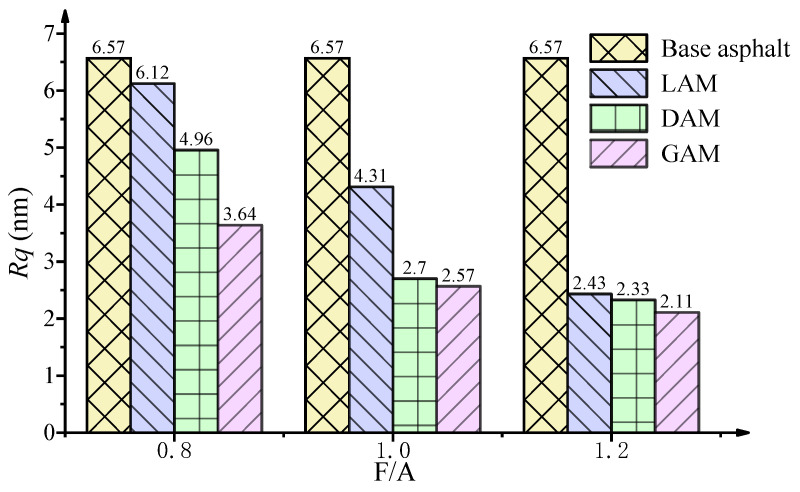
Results of the *Rq* surface roughness.

As shown in Figure 11, the base asphalt binder exhibited the highest value of *Rq* when compared with the three asphalt mastics for different F/A ratios. As the filler acidity increased, the values of *Rq* of the three asphalt mastics exhibited the same decreasing tendency at the same F/A ratio. As the F/A ratio increased, the surface roughness of each asphalt mastic also gradually decreased. For example, the *Rq* of LAM dropped from 6.12 to 2.43 with the increase of the F/A ratio from 0.8 to 1.2.

In conclusion, the indexes of the bee phase area, surface height, and surface roughness were shown to be effective in quantifying the micro-morphological characteristics of asphalt mastics, which could also reflect the effect of the asphalt–filler interaction on the phase morphologies of asphalt mastics.

### 3.3. Analysis of GRA

**Figure 12:**
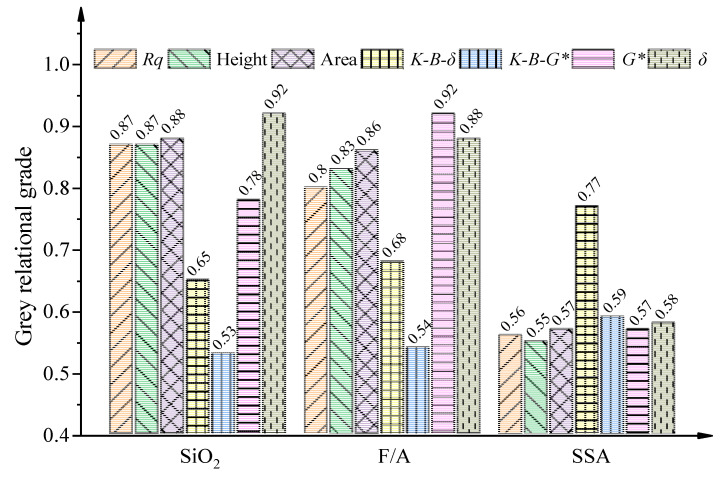
Grey relational grade.

To quantify the correlation of mineral fillers with the macro-rheological properties, asphalt–filler interaction, and micro-morphological characteristics of asphalt mastics, the GRA was performed according to the procedures described in Section 2.2.5. In the GRA model, the SiO_2_ content, F/A ratio, and SSA of the mineral fillers were the comparative sequences. The micro-morphological indexes (i.e., *Rq*, height, and bee phase area), macro-rheological indexes (G* and δ), and interaction ability indexes (*K-B-*δ and *K-B-*$G^{*}$) were the reference sequences, in which the G*, *δ*, *K-B-*δ, and *K-B-*$G^{*}$ were collected at 10 Hz during the frequency sweep tests. The original data series for the GRA are shown in Table 4 and the grey relational grade results are presented in Figure 12.

As shown in Figure 12, the SiO_2_ content and F/A ratio showed higher grey relational grade values with the macro–micro properties of asphalt mastics but with lower values for the asphalt–filler interaction ability. In contrast, the SSA had a greater effect on the asphalt–filler interaction ability when compared with the SiO_2_ content and the F/A ratio. Therefore, extra attention should be paid to the SiO_2_ content and F/A ratio when analyzing the macro–micro properties of asphalt mastics. Meanwhile, the SSA was the most important factor influencing the asphalt–filler interaction ability. No matter which factors were considered, the grey relational grades of the macro-rheological properties and micro-morphological indexes followed the same trend, which indicated that there was a certain relationship between the microscopic morphological features and the macroscopic rheological properties.

Furthermore, as for the micro-morphological indexes, the area of the bee phase had a better grey relational grade when compared to the indexes of height and *Rq*. Therefore, it is appropriate to use the bee phase area index to evaluate the micro-morphological characteristics of asphalt mastics. With further comparison, it was found that the grey relational grade of *K-B-*δ was generally greater than *K-B-*$G^{*}$, regardless of the SiO_2_ content, F/A ratio, and SSA of the mineral fillers, which indicated that the *K-B-*δ index was more sensitive to the filler properties than the *K-B-*$G^{*}$ index. Therefore, the *K-B-*δ index is recommended as a more appropriate index for evaluating the asphalt–filler interaction ability.

## 4. Conclusions

In this study, a series of macro-rheological tests and micro-morphological observations of asphalt mastics were carried out to investigate the characteristics and mechanisms of the asphalt–filler interaction and to identify the correlation between the properties of mineral fillers and macro–micro performances of asphalt mastics based on a grey relational analysis. The main conclusions are presented as follows:

1. The asphalt–filler interaction ability could be effectively evaluated using the *K-B-*δ index and *K-B-*$G^{*}$ index. Furthermore, the values of *K-B-*δ and *K-B-*$G^{*}$ presented an ascending trend with the increase of filler contents for the three asphalt mastics.
2. The interaction between the asphalt binder and filler was mainly caused by physical action since no obvious new absorption peaks appeared in the FTIR spectra.
3. The limestone filler, with its rough and uneven microsurface, exhibited the strongest asphalt–filler interaction ability in comparison with the diabase and granite fillers.
4. The micro-morphological characteristics of the asphalt mastics were susceptible to the acidity and content of the mineral fillers, which was reflected by the adsorption of the mineral fillers for polar fractions and the dispersion effect of the mineral fillers on wax crystals in the asphalt binder in AFM images. Furthermore, the effect of the asphalt–filler interaction on the micro-morphological differences in the asphalt mastics were quantitatively analyzed using the micro-morphological indexes, namely, the bee phase area, surface height, and surface roughness.
5. Through the GRA, the filler acidity and content exhibited a greater influence on the macro–micro properties of the asphalt mastics, while the SSA of the filler particles has a greater impact on the asphalt–filler interaction ability. Furthermore, the *K-B-*δ index is recommended as a more appropriate index for evaluating the asphalt–filler interaction ability.

## Figures and Tables

**Table 1:** Basic properties of the base asphalt binder.

Property	Unit	Result	Test Method
Penetration (100 g, 5 s, 25 °C)	0.1 mm	65.2	ASTM D 5
Softening point	°C	47.5	ASTM D 36
Ductility (5 cm/min, 15 °C)	cm	145.0	ASTM D 113
Viscosity (135 °C)	Pa·s	0.593	ASTM D 4402

**Table 2:** Properties of mineral fillers.

Property	Unit	Limestone	Diabase	Granite	Test Method
Apparent density	g/cm^3^	2.73	2.88	2.76	T0352-2005
SiO_2_ mass fraction	%	33.58	54.77	65.81	-
Specific surface area (SSA)	m^2^/g	8.27	6.39	1.65	T19587-2004

**Table 3:** Basic properties of asphalt mastics.

Mastic	F/A	Filler Volume Fraction (%)	Softening Point (°C)	Penetration at 25 °C (0.1 mm)	Ductility at 15 °C (cm)	Viscosity at 135 °C (Pa·s)
LAM	0.8	23.2	56.2	34.4	6.9	1.54
	1.0	27.4	57.8	32.9	5.1	2.03
	1.2	31.2	59.1	29.2	4.1	5.37
DAM	0.8	22.3	55.7	33.9	6.6	1.45
	1.0	26.4	57.7	32.1	4.8	1.89
	1.2	30.0	60.1	29.8	4.0	2.88
GAM	0.8	23.0	55.3	32.8	13.6	1.24
	1.0	27.2	57.5	30.5	5.9	1.31
	1.2	30.9	60.0	26.6	4.2	1.81

**Table 4:** The original data series of the grey relational analysis (GRA).

Mastic	Reference Sequences							Comparative Sequences		
	*Rq* (nm)	Height (nm)	Area	K-B-δ	$K\text{-}B\text{-}G^{*}$	G* (MPa)	*δ* (°)	SiO_2_ (%)	F/A	SSA (m^2^/g)
0.8 GAM	3.64	39.8	1123	0.09	0.04	6.22	65.66	65.81	0.8	1.649
1.0 GAM	2.57	27.8	987	0.20	0.10	6.47	64.62	65.81	1.0	1.649
1.2 GAM	2.11	14.8	707	0.33	0.52	9.05	63.10	65.81	1.2	1.649
0.8 DAM	4.96	47.2	1212	0.22	0.35	7.39	64.74	54.77	0.8	6.393
1.0 DAM	2.70	35.1	1186	0.26	0.38	7.76	64.22	54.77	1.0	6.393
1.2 DAM	2.30	24.4	778	0.54	0.68	10.01	61.30	54.77	1.2	6.393
0.8 LAM	6.12	51.5	1425	0.47	0.56	8.39	62.93	33.58	0.8	8.274
1.0 LAM	3.12	41.7	1280	0.68	0.71	9.64	60.99	33.58	1.0	8.274
1.2 LAM	2.43	31.5	857	1.05	0.74	10.70	56.68	33.58	1.2	8.274
